# An Unhappy Juxtaposition

**Published:** 1891-03-21

**Authors:** 


### Passing Topics.

### AN UNHAPPY JUXTAPOSITION.

It is really too bad of that usually good friend of the hospitals, the *Daily Telegraph*, to include the proceedings of the Local Government Board concerning the "alleged scandals" at the Eastern Hospital, Homerton, in the same paragraph and under the same heading as the inquiry by the Select Committee of the House of Lords into the management of the metropolitan hospitals.

There is all the difference in the world between the two inquiries, as everybody knows, or, rather, as everybody ought to know. They possess nothing in common, but there is just enough similarity, as regards the subject-matter, to hopelessly confuse that ubiquitous and somewhat dunder-headed person, the superficial reader and thinker. What, most people would ask, is a newspaper for, but to purvey news and to convey impressions which are to be taken unquestioningly? In these days of drive and hurry a man swallows his news by paragraphs, whole, and gulps down opinions, social and political, just as his favourite journal offers them, and if he should read under the headline of "Metropolitan Hospitals" the evidence of a witness that the butter was "bad," the eggs "rotten," the fish "always haddock and always stinking," the meat "invariably boiled mutton"—like old Cheesman's, and that's our putting in—and as "invariably tainted," that the milk was "sour four days out of seven," and that "he had seen three children put into the same bath together," all of which choice observations are culled from a single record, it is much more than probable he will fail to distinguish between what is going on at the offices of the Metropolitan Asylums Board and the proceedings which are taking place in the precincts of St. Stephens. He will not unnaturally conclude that this wholesale condemnation applies to one or all of the veritable hospitals of London and that the sooner he withdraws his subscription, supposing him to give any, the better.

The average reader is slow to correct anything he sees in print, and he will have no difficulty about believing and asserting that the most damaging evidence has been brought home to those great institutions which have ministered to hundreds of thousands of poor suffering folk with scarcely a serious charge of neglect or shortcoming within the memory of living man. If we understand the matter rightly, what charges there are against the voluntary hospitals point to profusion, not shortcoming; and, put shortly, was it not some notion that they were too benevolent which pervaded the minds of the chief promoters of the Lords' Inquiry?

But the power of discrimination has ever been a rare individual faculty, and under the conditions of modern life it grows rarer day by day. If public journals show no greater possession of it than the *Daily Telegraph*, it is inevitable that the public mind will become hopelessly confused, and grave injustice would be done to institutions which, whatever their other imperfections, will not be found wanting in the sympathy for suffering and earnestness of philanthropic purpose which would redeem a much less capable administration, and render altogether impossible the coarser concomitants of the more perfunctory *régime* of many of our rate-supported infirmaries.

It is an unfortunate circumstance that the two inquiries should be proceeding simultaneously, and that the echoes of each should be mingling in the air. The voluntary hospitals have nothing to gain, and everything to lose, by complications and misunderstandings. Even those who fully recognize the distinction between the two inquiries will find it difficult to keep the threads of both quite separate and untangled in their minds. We look for something more capable and intelligent at the hands of a great daily paper than the jumble we have commented upon, and we must hope it does not foreshadow the kind of criticism which is to be let loose when the Lords have finished their labours. Agricola.

